# The Boss' Healthy Buddies Nutrition Resource Is Effective for Elementary School Students

**DOI:** 10.1155/2018/4659874

**Published:** 2018-05-02

**Authors:** David W. Pittman, Ida R. Bland, Isai D. Cabrera, Kassie E. Franck, Emily L. Perkins, Nicole A. Schmidt, Heather N. Allen, Savanah R. Atkins, Sharyn B. Pittman

**Affiliations:** Department of Psychology, Wofford College, 429 North Church Street, Spartanburg, SC 29303, USA

## Abstract

Previously we have shown that our Healthy Eating Decisions school-based intervention can influence students' selections of the healthiest foods available in their elementary school cafeterias through positive reinforcement techniques. Although effective, we recognized that students were missing fundamental nutrition knowledge necessary to understand why the Healthy Eating Decisions program identified particular beverages and foods as the healthiest in the cafeteria. Therefore, we developed the Boss' Healthy Buddies nutrition education resource as a freely available curriculum matched with South Carolina education standards and designed for elementary school students from kindergarten through fourth grade. The current study implemented Boss' Healthy Buddies and compared its efficacy to a commercially available nutrition program, CATCH. Elementary school students in Spartanburg, South Carolina, received weekly twenty-minute Boss' Healthy Buddies lessons for eight weeks. Results from preassessment and postassessment surveys were compared with a positive control elementary school using the CATCH program and a negative control school receiving no nutrition education. Results show that Boss' Healthy Buddies was equally effective as the CATCH program in improving the nutrition attitudes regarding healthiest beverages and food selections with the advantage of being freely available and minimizing the impact on classroom instruction time. In order to reduce most effectively the high prevalence of childhood overweight and obesity, it is crucial that children are taught nutrition education to support healthy eating habits at an early age. Both the Healthy Eating Decisions school-based intervention and the Boss' Healthy Buddies nutrition education program are available online for use as free resources to aid in reducing childhood overweight and obesity within elementary schools.

## 1. Introduction

In the United States, the rates of childhood obesity have more than tripled since 1971. In the elementary school population aged 6 to 11 years old, the prevalence of childhood obesity has risen from 11.3% in 1988–1994 to 19.6% in 2007-2008 [1]. By the end of the 2000s, increased public awareness of this obesity epidemic coupled with local and national movements [2] to prevent and reduce childhood obesity had stabilized the childhood obesity rate around 17.5% (2013-2014) to 18.4% (2015-2016) but showed little to no signs of decreasing the childhood obesity prevalence [1, 3]. Overweight and obese children are at risk for numerous health problems [4–6], negative psychosocial consequences, and lower school performance [7, 8]. Obese girls were reported to be 1.51 times more likely to be held back a grade and 2.9 percent more likely to self-report being a poor academic student. While obese boys were 1.46 times more likely to self-report being a poor student and 2.18 times more likely to drop out of school before their normal weight peers [9]. In addition to lower academic performance, childhood obesity also leads to behavioral issues with low self-esteem [10] and increased aggressive and destructive behaviors [11]. Furthermore, 70% of obese children persist into becoming obese adults [12, 13].

Early prevention and intervention strategies are necessary to combat these poor developmental outcomes. The potential impact of the school environment to reduce childhood obesity rates is promising. Pyle et al. [8] suggest a multifaceted approach including education on nutrition and healthy lifestyle behaviors targeting the youngest students as the best tactic for schools to address childhood obesity. A longitudinal study supports this multifaceted school-based approach reporting positive changes in BMI and academic performance in elementary school children following interventions that included healthier food options, education on healthy lifestyle choices, and increased physical activity [14]. Previously, we developed and provided evidence-based effectiveness of a school-based intervention to increase elementary students' selection of the healthiest food and beverage items during their cafeteria lunches [15]. Our program, Healthy Eating Decisions, identified the “healthiest” combination of entrée and side items on each daily lunch menu using a computer algorithm based on the DASH eating plan developed by the U. S. National Institutes of Health [16]. Since the original implementation of the Healthy Eating Decisions program in Spartanburg County, South Carolina elementary schools, we have tracked the longitudinal success from 2009 to 2015 of the program to continue to promote increased selection of the identified “healthiest” lunch items [17]. However, our continued involvement in the elementary school environment led us to recognize that many students lacked the fundamental nutrition knowledge necessary in order to understand how and why certain beverage and food items were considered the healthiest combination during their daily lunches. In spite of mandated weekly nutrition education by the South Carolina state legislature [18], we witnessed a gap in effective nutrition education programs missing from the elementary school curriculum. This problem in unfunded legislative mandates to provide nutrition education is not limited to the state of South Carolina. In fact, all but four U. S. states have some form of nutrition education policy but few policies are being actively implemented [19]. The two greatest barriers to implementing nutrition education curriculum are a lack of classroom time for additional curricular components beyond the mandated state standards and the cost of commercially available nutrition education programs.

In this study, we present a novel and innovative approach to overcome both of these barriers by integrating nutrition education into the South Carolina state education standards curriculum in a freely available nutrition education resource. Targeting kindergarten through fourth grade, our nutrition education resource called Boss' Healthy Buddies includes 35 modules (one for each week of the elementary school year) covering topics such as the importance of nutrition and physical activity, general information of certain foods and their proper proportions and daily servings, and the difference between “Go,” “Slow,” and “Whoa” foods. Through our modular nutrition education resource, teachers utilize in-class exercises reinforcing state standards while at the same time providing much needed nutrition knowledge that can influence and shape the development of healthy habits in children at a young age. Boss' Healthy Buddies also reaches beyond the classroom with at-home activities called Backpack Bulletins that engage the students' families in each themed module. In order to test the effectiveness of the Boss' Healthy Buddies nutrition education resource, this study included three Spartanburg County elementary schools: one school that received Boss' Healthy Buddies (intervention school), another that received a commercially available nutrition education program called CATCH [20] (positive control school), and a third school providing no nutrition education programming (negative control school). Nutrition attitudes regarding beverages and foods as well as daily eating habits of the elementary students were assessed at the beginning of the school year and immediately following an eight-week intervention period. The aim of this study was to show that the Boss' Healthy Buddies resource was at least equally effective as the commercially available nutrition education resource and that employing any nutrition education program was superior to not providing nutrition education within the elementary school environment.

## 2. Materials and Methods

### 2.1. Study Design and Participants

Participants included a convenience sample of 1,710 students from kindergarten to fourth grade attending three public elementary schools in Spartanburg, SC, on the preassessment and postassessment testing days. Each of the three elementary schools had demographics similar to the adult population of their resident county. Both a within-subject design (preassessment/postassessment surveys) at the intervention and positive control schools and a between-subject design (within preassessment and postassessment surveys) across all three schools were used to measure the effectiveness of the nutrition education programs. The intervention school included 520 students (45% female, 55% male; 59% Caucasian, 35% African American, 4% Hispanic, 2% others; 48% free or reduced school lunch), the positive control school included 615 students (46% female, 54% male; 85% Caucasian, 7% African American, 3% Hispanic, 5% others; 41% free or reduced school lunch), and the negative control school included 575 students (47% female, 53% male; 5% Caucasian, 87% African American, 6% Hispanic, 2% other; 86% free or reduced school lunch). The specific schools were selected based on the criteria of either not having had previous nutritional education programs or the availability of the CATCH nutrition program and willingness of the principal to participate. All aspects of this study were approved by the Institutional Review Board of Wofford College and the principals of each elementary school.

### 2.2. Intervention

All elementary students at the positive control school had previously received two years of weekly instruction from the CATCH nutrition education resource that is commercially available in Flaghouse [20]. The weekly CATCH nutrition education instruction continued during this study's eight-week intervention period. Previously, the CATCH curriculum has been shown to be effective at improving student knowledge of healthy eating behaviors and increasing healthy food selection by those students [21–23]. A major disadvantage of the CATCH curriculum is its financial and classroom instruction time cost to implement in elementary schools. Students in all kindergarten through fourth grade classes at the intervention school received weekly twenty-minute lessons from the Boss' Healthy Buddies nutrition education resource for eight consecutive weeks. The module “Why it is important to be Healthy?” was the first lesson taught in all classes which was followed the second week with instruction using the module “5-2-1-0.” For intervention weeks three through eight, random modules were selected from the 35-module library of Boss' Healthy Buddies nutrition education lessons. The researchers verified fidelity to the curriculum with weekly contact and recording of which lesson was taught for that week. Modules from the Boss' Healthy Buddies curriculum can be viewed and downloaded online at http://healthyeatingdecisions.com/bossbuddies.

### 2.3. Outcome Measures

The School Physical Activity and Nutrition (SPAN) questionnaire was developed through support by the Centers for Disease Control and the U. S. Department of Agriculture as an assessment tool of physical activity, nutrition attitudes, and daily food eating behaviors in children [24–26]. In this study, we used a modified version of the SPAN assessment tool focusing on daily eating behaviors and nutrition attitudes regarding beverages and foods [27]. The 21-item questionnaire was administered in the second week of the school year prior to the eight-week intervention period of Boss' Healthy Buddies or CATCH instruction (preassessment). Weekly instruction with the Boss' Healthy Buddies and CATCH resources began the week after the preassessment, and the week immediately following the eight-week intervention period, the questionnaires were administered again (postassessment). Students in second through fourth grade completed a written version of the assessment, while students in kindergarten and first grade completed an oral assessment that was videotaped so that responses could be quantified following the classroom session.

### 2.4. Data Analysis

The questionnaire items were grouped into three categories: daily eating habits, nutrition attitudes regarding beverages, and nutrition attitudes regarding foods. The responses to each questionnaire item were summated for each grade at each school and were normalized to percentages. These percent responses were subjected to Wilcoxon Signed Rank tests to identify significant (*p* < 0.05) differences between the preassessment and postassessment response rates within each experimental condition, and Kruskal–Wallis tests were used to compare the response rates across the three experimental conditions within the preassessment and postassessment response rates. Post hoc pairwise comparisons using Mann–Whitney *U* tests identified significant differences between specific experimental conditions in the postassessment response rates.

## 3. Results

Analysis of the preassessment data revealed no significant differences in the response rates across the three experimental conditions for any of the questionnaire items indicating that before the education period, the students at all three schools displayed similar daily eating habits and nutrition attitudes towards beverages and foods. Interestingly, the students at the positive control school who had previously received two years of CATCH instruction showed no significant differences in their preassessment response rates to any item on the questionnaire when compared with the negative control and intervention schools.

### 3.1. Daily Eating Habits

Participants were asked how many servings (zero to five) of fruit, vegetables, meats, breads, and desserts they consume on most days. The only effect of any of the experimental conditions on daily eating behaviors was for the number of daily desserts consumed. Within the intervention school, there was a significant decrease, *z*=4.153, *p*=0.043, in the percent of participants consuming two daily desserts from the preassessment (23%) to the postassessment (15%) measures with a concomitant increase in the response for zero to one daily dessert serving (47% to 57%). There was also a between-subject main effect of school, *χ*^2^ (2, 15) = 6.080, *p*=0.048, showing a greater percent of participants who consumed zero to one daily dessert at both the intervention school (57%) and the positive control school (52%) compared to the negative control school (30%). The selective effect on the eating behaviors for desserts compared to all other foods demonstrates the ability of any nutrition education program to have a positive influence on the unhealthiest of the eating behaviors.

### 3.2. Nutrition Attitudes Regarding Beverages

**Figure 1:**
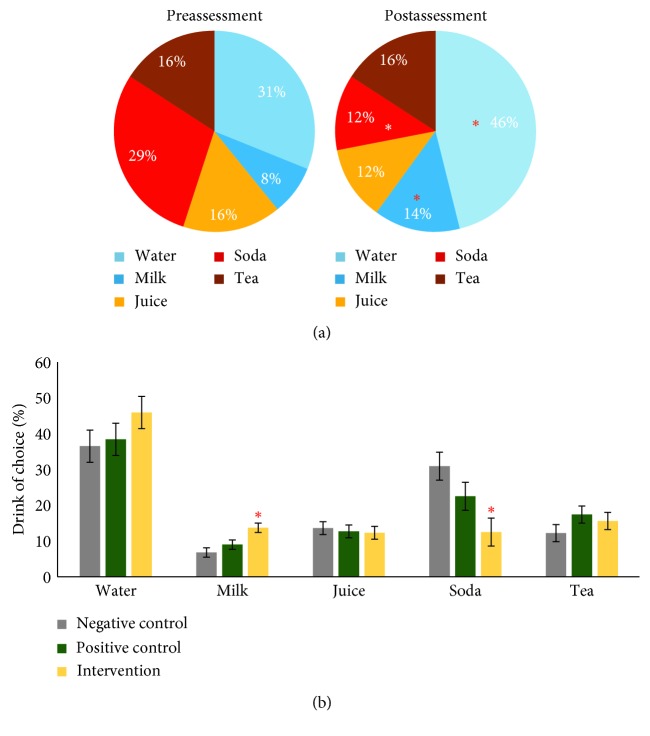
Percent change in preferred beverage within the intervention school (a) and between the three experimental conditions (b). Single star indicates *p* < 0.05 significant differences in drink choice between preassessment and postassessment (a) or significant differences between the experimental conditions within a drink type (b).

Participants were asked which of the five following options was their preferred drink of choice: water, milk, juice, soda, or tea. There was a significant shift in the percent responses within the intervention school between the preassessment and postassessment. As shown in Figure 1(a), significantly more participants chose either water, *z*=2.023, *p*=0.043, or milk, *z*=2.023, *p*=0.043, following the eight-week intervention with a concomitant decrease in soda, *z*=2.023, *p*=0.043, as their preferred beverage.

As shown in Figure 1(b), comparing across the three experimental conditions revealed a main effect of school due to an increase in the percent of intervention participants selecting milk, *χ*^2^ (2, 15) = 8.520, *p*=0.014, and a decrease in the percent of intervention participants selecting soda, *χ*^2^ (2, 15) = 6.260, *p*=0.044, compared to participants in both the negative and positive control schools. Post hoc Mann–Whitney tests revealed a significant increase in milk as the drink of choice at the intervention school compared to both the negative control, *z*=2.611, *p*=0.009, and the positive control, *z*=2.095, *p*=0.036. The significant effect on soda was attributed to a significant decrease for the intervention school compared to the negative control, *z*=2.193, *p*=0.028.

**Figure 2:**
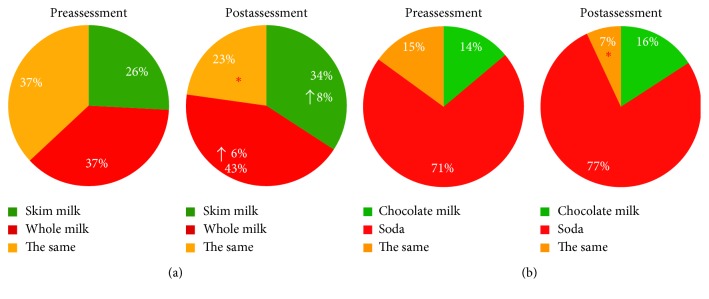
Percent change in nutrition attitudes regarding beverage selections within the intervention school for skim versus whole milk (a) and the amount of sugar in chocolate milk versus soda (b). Single star indicates *p* < 0.05 significant differences between preassessment and postassesment.

There was a significant within-subject effect of the intervention on the percent of participants responding to whether skim or whole milk were equally or differentially healthy. As shown in Figure 2(a), significantly fewer participants reported that skim and whole milk were equally healthy after the intervention, *z*=2.023, *p*=0.043. The intervention also significantly decreased the percent of participants responding that chocolate milk and soda had the same amount of sugar, *z*=2.023, *p*=0.043, as shown in Figure 2(b).

### 3.3. Nutrition Attitudes Regarding Foods

**Figure 3:**
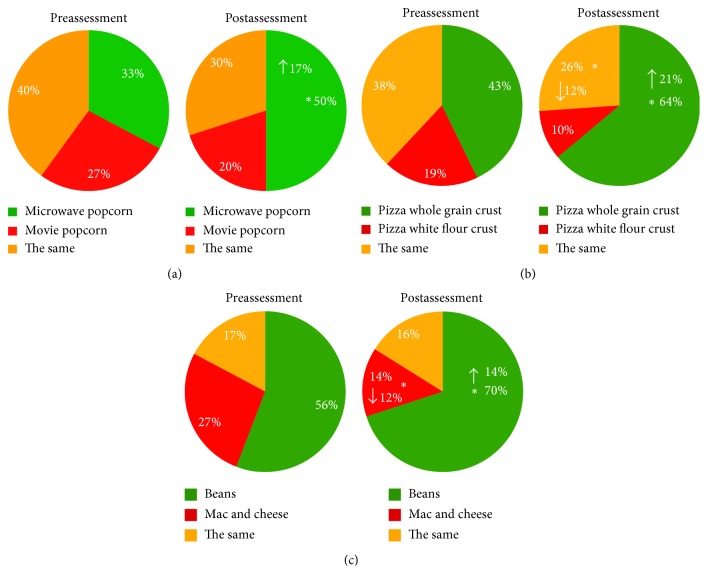
Percent change in nutrition attitudes regarding which foods are healthier within the intervention school for microwave popcorn versus movie popcorn (a), whole grain versus white flour pizza crust (b), and beans versus mac and cheese (c). Stars indicate *p* < 0.05 significant differences between preassessment and postassesment measurements.

As shown in Figure 3, participants within the intervention school were asked to choose the healthier option out of two food choices or to indicate if the food choices were equal in healthiness. In Figure 3(a), there was a significant 17% increase in the correct response of microwave popcorn as the healthiest option, *z*=2.023, *p*=0.043, when compared to movie popcorn.  Figure 3(b) compares pizza with whole grain crust to pizza with white flour crust revealing a significant 21% increase in the percent of participants choosing pizza with whole grain crust as healthiest paired with a significant 12% decrease in the incorrect response that the two pizzas are equally healthy, both *z*=2.023, *p*=0.043. There was a significant 13% decrease in the incorrect response of mac and cheese coupled with a significant 14% increase in beans as the healthier choice following the eight-week nutrition education intervention (Figure 3(c); both *z*=2.023, *p*=0.043).

**Figure 4:**
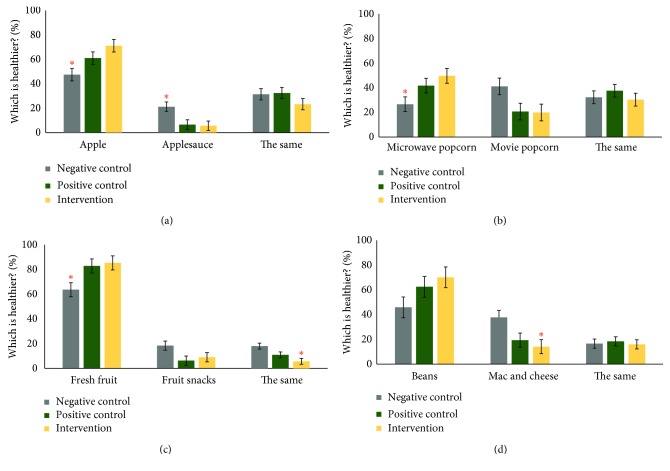
Percent change in nutrition attitudes regarding which foods are healthier across the three experimental conditions for apple versus applesauce (a), microwave popcorn versus movie popcorn (b), fresh fruit versus fruit snacks (c), and beans versus mac and cheese (d). Single star indicates *p* < 0.05 significant differences.

As shown in Figure 4, the between-subject analysis of the three experimental conditions revealed consistent and similar positive influences on nutrition attitudes across foods for both nutrition education conditions, Boss' Healthy Buddies (intervention), and CATCH (positive control) as compared to no nutrition education (negative control). As shown in Figure 4(a), there was a main effect of school for the percent choosing apple, *χ*^2^ (2, 15) = 7.280, *p*=0.026, and applesauce, *χ*^2^ (2, 15) = 6.660, *p*=0.036, as the healthiest option with the intervention school choosing apple significantly more than the negative control school, *z*=2.402, *p*=0.016, and the negative control school choosing applesauce significantly more than both the positive control, *z*=1.984, *p*=0.047, and intervention schools, *z*=2.402, *p*=0.016.

Demonstrating the importance of nutrition education, both the positive control and intervention schools chose microwave popcorn (Figure 4(c); *χ*^2^ (2, 15) = 7.473, *p*=0.024) and fresh fruit (Figure 4(d); *χ*^2^ (2, 15) = 7.940, *p*=0.019) as the healthier options significantly more than the negative control school. The intervention school also had the least percentage of responses that fruit snacks were equally healthy as fresh fruit, *χ*^2^ (2, 15) = 10.220, *p*=0.006. Finally, there was a main effect of school for mac and cheese compared to beans, *χ*^2^ (2, 15) = 6.500, *p*=0.039, with the intervention school choosing mac and cheese as the healthier option significantly less than the negative control school (Figure 4(d)). The similarities in results between the intervention and positive control school paired with consistent differences with the negative control school results suggest that any nutrition education program is better than no nutrition education and that Boss' Healthy Buddies appears to be equally effective as CATCH in positively influencing nutrition attitudes.

**Figure 5:**
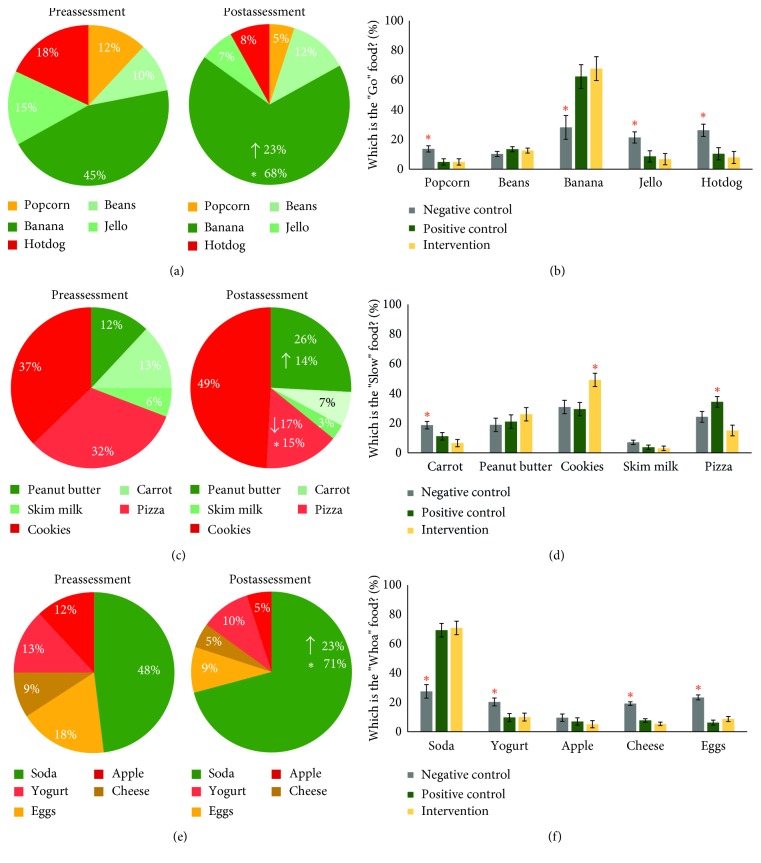
Percent change in nutrition attitudes regarding correctly identifying “Go” (a and b), “Slow” (c and d), and “Whoa” (e and f) food items within the intervention school (a, c, and e) and across all three experimental conditions (b, d, and f). Stars indicate *p* < 0.05 significant differences.

Teaching students to label foods as “Go,” “Slow,” and Whoa” is a consistent theme across most nutrition education curricula including both CATCH and Boss' Healthy Buddies. “Go” foods are foods that can be eaten readily and have the most nutritional value while “Slow” foods should not be eaten as readily as “Go” foods but do contain some nutritional value compared to “Whoa” foods which should be avoided and are the least healthy. In Figure 5, the left-side panels show changes in identifying “Go” (Figure 5(a)), “Slow” (Figure 5(c)), and “Whoa” (Figure 5(e)) foods from preassessment to postassessment within the intervention school. There were significant increases in the selection of banana, the correct “Go”, *z*=2.023, *p*=0.043, and soda, the correct “Whoa”, *z*=2.023, *p*=0.043, food items and a significant decrease for pizza, an incorrect “Slow” food, *z*=2.023, *p*=0.043, indicating effectiveness of the Boss' Healthy Buddies program to teach correct labeling of healthy and unhealthy foods.

As shown in the right-side panels of Figure 5, there were significant differences in identifying “Go,” “Slow,” and “Whoa” foods between the negative control school and the positive control and intervention schools, which did not differ from each other except for the “Slow” food responses of cookies and pizza. In Figure 5(b), there were significant differences for popcorn, *χ*^2^ (2, 15) = 6.627, *p*=0.036, banana, *χ*^2^ (2, 15) = 6.740, *p*=0.034, jello, *χ*^2^ (2, 15) = 6.020, *p*=0.049, and hotdog, *χ*^2^ (2, 15) = 6.320, *p*=0.042. The negative control school choose the correct “Go” food, banana, significantly less than both the positive control school, *z*=2.488, *p*=0.013, and intervention school, *z*=2.611, *p*=0.009. The negative control school also differed from both the positive control and intervention schools in incorrectly identifying popcorn, *z*=2.193, *p*=0.028 (both statistics), jello, *z*=1.984, *p*=0.047 (positive) and *z*=2.193, *p*=0.028 (intervention), and hotdog, *z*=1.984, *p*=0.047 (positive) and *z*=2.402, *p*=0.016 (intervention).

For the “Whoa” foods shown in Figure 5(f), there were significant differences for soda, *χ*^2^ (2, 15) = 9.500, *p*=0.009, yogurt, *χ*^2^ (2, 15) = 6.860, *p*=0.032, cheese, *χ*^2^ (2, 15) = 11.015, *p*=0.004, and eggs, *χ*^2^ (2, 15) = 9.500, *p*=0.009. The negative control school choose the correct “Whoa” food, soda, significantly less than both the positive control school and intervention school, *z*=2.611, *p*=0.009 (both statistics). The negative control school also differed from the positive control and intervention schools in incorrectly identifying yogurt, *z*=2.193, *p*=0.028 (both statistics), cheese, *z*=2.611, *p*=0.009 (both statistics), and eggs, *z*=2.611, *p*=0.009 (both statistics).

The “Slow” food category is a more difficult concept for elementary students to understand than “Go” and “Whoa” foods. Within the intervention school, shown in Figure 5(c), there was a significant decrease in selecting the incorrect pizza response, *z*=2.023, *p*=0.043, and a 14% increase in the correct selection of peanut butter that did not reach significance, *z*=1.753, *p*=0.080. Similarly, examining the responses across the three experimental conditions revealed confusion about the correct “Slow” food, peanut butter, as shown in Figure 5(d). There were significant effects for the incorrect “Slow” food responses of carrot, *χ*^2^ (2, 15) = 7.428, *p*=0.024; negative control > intervention, *z*=2.611, *p*=0.009; cookies, *χ*^2^ (2, 15) = 6.136, *p*=0.0047; intervention > negative control, *z*=1.984, *p*=0.047, and positive control, *z*=2.193, *p*=0.028; and pizza, *χ*^2^ (2, 15) = 7.440, *p*=0.024; positive control > negative control, *z*=2.402, *p*=0.016, and intervention, *z*=2.193, *p*=0.032.

## 4. Discussion

The aim of the current study was to assess the effectiveness of the Boss' Healthy Buddies nutrition education resource that minimizes the impact on classroom instruction time and provides a free alternative to the commercially available programs. The results provide evidence that the Boss' Healthy Buddies nutrition education resource is more effective than no nutrition education and equally effective as the commercially available CATCH resource in influencing healthier daily eating habits and positive nutrition attitudes towards healthier beverage and food selections.

### 4.1. Daily Eating Habits

Both Boss' Healthy Buddies and CATCH successfully increased the percent of participants reporting only zero or one daily dessert compared to the no nutrition education condition in which the majority of responses (41%) reported eating behaviors of five daily servings of desserts. The lack of significant effects for either Boss' Healthy Buddies or CATCH to shift eating behaviors for fruits, vegetables, breads, or meats could be due to the limited time frame of the eight-week intervention period. Within eight weeks, students clearly learned that limiting desserts, the most unhealthy food category, was important; however, the more nuanced appropriate servings of healthier foods such as fruits, vegetables, breads, and meats may require instruction and repetition over a longer implementation period. Boss' Healthy Buddies has 35 modules, one for each week of the elementary school year; therefore, with continued use of the nutrition education resource throughout the school year, we may see influences on other eating behaviors in the planned year-end follow-up assessments.

### 4.2. Nutrition Attitudes

Results from the preassessment/postassessment within-subject design at the intervention school clearly show increased awareness of elementary students as to which beverages and foods are healthier after the eight-week education period. There were significant shifts towards better understanding of which choices are healthier across multiple food categories including beverages, snack foods (popcorn), and entrées (mac and cheese, beans, and pizza). When comparing across postassessment measures, the eight-week implementation of the Boss' Healthy Buddies program was equally effective as the CATCH resource at the positive control school, and the intervention school showed greater gains in correctly identifying milk as a drink of choice instead of soda (Figure 1(b)). Strikingly, the robust unhealthy selections across beverage and food selections by participants at the negative control school underscore the importance of providing any form of nutrition education to elementary students.

### 4.3. Study Limitations

A potential limitation of research in public schools is the inability to control for student absences during either the assessment days or the days on which the interventions were taught during the eight-week period. Convenience sampling is commonly utilized in these types of school-based intervention studies, and the relatively small number of student absences on any given day is unlikely to skew the data. Furthermore, differences in the convenience-sampling population influencing the data would be unusual to produce the similar gains in the postassessment measures evidenced for both the positive control and intervention schools.

Surprisingly, analysis of preassessment data did not reveal increased healthier eating habits or nutrition attitudes for the positive control school that for the previous two years had been implementing the CATCH curriculum. Fatigue effects in which potential gains in healthy behavior may have been observed early in the implementation of the CATCH curriculum and waned over the two years are unlikely here as there were similar gains in healthy behaviors for both the positive control and intervention schools on the postassessment measures following the eight-week intervention period. A more plausible explanation could be that potential gains in healthy behavior achieved by the CATCH program are lost over the three-month summer break resulting in a return to baseline at the start of each school year. If this is the case, then it is even more important to maintain the repetition of nutrition education resources in as many years as possible across all grades in elementary school. It may also point to the importance of influencing healthy eating behaviors at home as well as in the school environment, which is the target of the Backpack Bulletins component of the Boss' Healthy Buddies resource.

One explanation of the gains in healthy behavior for the Boss' Healthy Buddies resource could be a novelty effect in which improvements on the postassessment measures were due to increased interest in the nutrition curriculum rather than actual gains in learning. Only future longitudinal testing will be able to clarify actual learning gains and novelty effects. It will be interesting to compare assessments of the Boss' Healthy Buddies resource at the start of the next school year with previous end of the school year assessments and the CATCH preassessment to see if similar losses in healthy behaviors occur during the summer break following a year-long implementation of the Boss' Healthy Buddies resource.

## 5. Conclusions

The Boss' Healthy Buddies resource was shown to be as effective as the commercially available CATCH resource but with two substantial advantages. One, the Boss' Healthy Buddies resource is freely available to any elementary school eliminating the financial barrier associated with most other comprehensive nutrition education programs. Second, valuable classroom instruction time is not impacted when implementing the Boss' Healthy Buddies resource due to grade-specific South Carolina state education standards being incorporated into each of the nutrition lessons. South Carolina is ranked seventh in the United States for obesity with approximately 18.2% of adolescents having an overweight classification and an additional 16.3% classified as obese in the latest report from 2015 [28]. There are 575,261 students enrolled in 1,307 South Carolina public elementary schools who could benefit from freely available nutrition education [29]. Our goal is to offer free access and assistance in implementing both the Healthy Eating Decisions program and the Boss' Healthy Buddies nutrition education resource in all elementary schools in South Carolina beginning in 2019. This short-term study is the first evidence demonstrating the effectiveness of the Boss' Healthy Buddies program to influence the nutrition attitudes and healthy eating habits of elementary school students with future longitudinal studies planned. Although implementing the Boss' Healthy Buddies resource is likely not sufficient alone to reduce childhood overweight and obesity, incorporating healthy nutrition education into the state standards taught in all grades of elementary school could be a very important step towards the goal of improving the health of the children of South Carolina. Our future directions include longitudinal measures of the effectiveness of the program as well as collecting body mass index data to track potential changes in the prevalence of overweight and obesity in schools implementing the Healthy Eating Decisions and Boss' Healthy Buddies programs. As Boss' Healthy Buddies is implemented in future schools, we will provide assessment tools to continue to monitor the effectiveness of the program. All materials necessary to successfully implement these programs including the assessment tool and nutrition education resource are freely available at http://healthyeatingdecisions.com/bossbuddies.

## Data Availability

The data set is available on request to the corresponding author.
